# Lung adenocarcinoma harboring complex EML4-ALK fusion and BRAF V600E co-mutation responded to alectinib

**DOI:** 10.1097/MD.0000000000030913

**Published:** 2022-10-07

**Authors:** Weihong Guo, Jianping Liang, Dandan Zhang, Xikun Huang, Yanhua Lv

**Affiliations:** a Zhongshan City People’s Hostipial, Zhongshan, Guangdong Province, China.

**Keywords:** alectinib, BRAF V600E, co-mutation, EML4-ALK, lung adenocarcinoma

## Abstract

The echinoderm microtubule-associated protein-like 4 gene and anaplastic lymphoma kinase gene (EML4-ALK) is the most frequent fusion variant of ALK rearrangements in non-small cell lung cancer (NSCLC). With the widespread application of next-generation sequencing (NGS), more fusions and co-mutations of EML4-ALK have been discovered. Complex co-mutation of EML4-ALK fusions together with BRAF V600E, though rarely occurred, also deserves attention to determine the standard of caring these patients. Herein, we report a case of lung adenocarcinoma harboring a complex ALK fusion that coexisted with a BRAF mutation, as tested by DNA-NGS prior to treatment.

**Patient concerns:**

A 51-year-old non-smoking man, without any symptoms, was admitted to hospital due to small pulmonary nodules and enlarged supraclavicu larlymph nodes found in health checkup.

**Diagnosis:**

He was diagnosed with stage IVB (T4N3M1c) lung adenocarcinoma. BRAF V600E (abundance 3.75%) mutation and a novel thus little-understood EML4-ALK (E13, A5; abundance 2.16%) fusion were identified by DNA-NGS analysis of lymph node biopsy tissue in December 2019.

**Interventions:**

Darafenib plus trametinib targeted therapy and chemotherapy were given firstly, but tumor progression was not inhibited. The ALK inhibitor alectinib was prescribed then.

**Outcomes:**

The patient exhibited a rapid disease response to ALK tyrosine kinase inhibitors alectinib with a complete remission of widespread metastatic disease and progression-free survival of more than 26 months, but not to darafenib plus trametinib targeted BRAF V600E therapy. Re-analyzed the patient’s DNA-NGS original data, showed it is a rare and complex EML4-ALK (E13, A5, A20) fusion in fact. Additional RNA-NGS analysis showed it verified to be a canonical EML4-ALK (E13, A20) fusion transcript and coexisting with a BRAF V600E mutation.

**Lessons:**

This case suggests that for patients with rare or complex EML4-ALK fusions at DNA level, additional RNA-NGS is necessary to verify its functionality as early as possible. Targeting EML4-ALK firstly may be more preferable despite the coexisting of BRAF V600E.

## 1. Introduction

Targeted therapy has revolutionized the treatment of non-small cell lung cancer (NSCLC) patients harboring mutations in driver genes. With the widespread application of next-generation sequencing (NGS), more fusions and co-mutations have been discovered, that play a critical role in accurate prescription of targeted drugs and predicting the therapeutic efficacy.^[1]^ Anaplastic lymphoma kinase (ALK) rearrangements and BRAF V600E mutations are rare genetic variants in NSCLC with incidences of about 5% and 2% respectively.^[2,3]^ Complex EML4-ALK fusion together with BRAF V600E co-mutation is more uncommon, and the standard of care is unclear. We report a case of lung adenocarcinoma harboring a complex EML4-ALK (E13, A5, A20) fusion that coexisted with a BRAF mutation, as tested by DNA-NGS prior to treatment.

## 2. Case presentation

**Figure 1.**
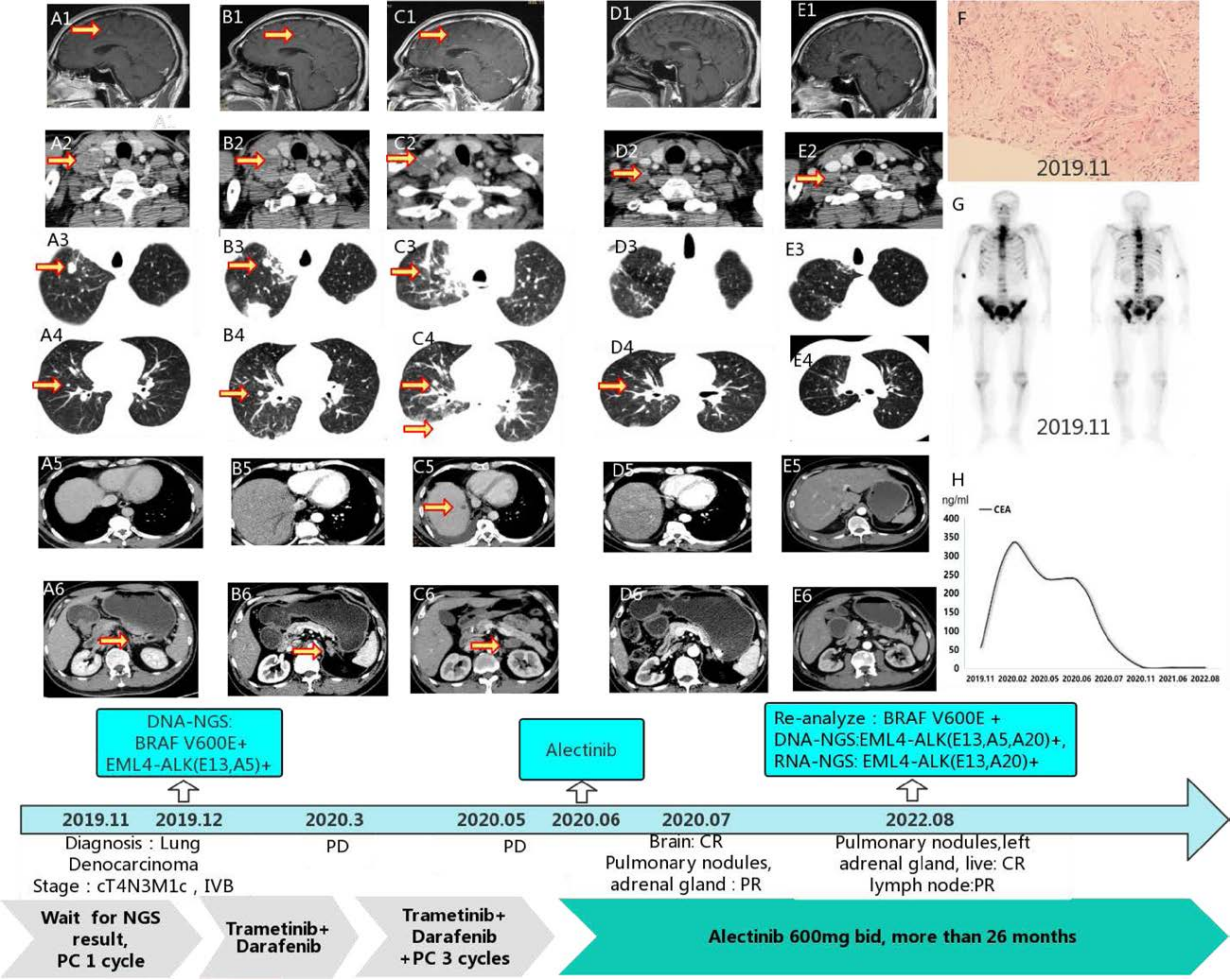
The clinical course of the diagnosis and treatment of the patient. CR = complete response, PD = progressive disease, PC = pemetrexed +  carboplatin, PR = partial response.

A 51 -year-old nonsmoking man, without any symptoms, was admitted to hospital in November 2019 due to small pulmonary nodules and enlarged supraclavicular lymph nodes found in health checkup. Biopsy of the supraclavicular lymph nodes revealed metastases from lung adenocarcinoma. Multiple metastases in lung, adrenal gland, brain and bone were also detected by computed tomography scan, magnetic resonance imaging and whole-body emission computed tomography. A clinical stage of T4N3M1c (stage IVB) lung adenocarcinoma was confirmed (Fig. 1).

Due to heavy tumor burden, chemotherapy with pemetrexed + carboplatin was started at once; meanwhile, lymph node biopsy tissue was sent for DNA-NGS testing, and results returned in December 2019. BRAF V600E mutation (abundance 3.76%) and EML4-ALK (E13, A5) (abundance 2.16%) fusion was reported; the latter was little reported and its clinical significance was unclear. We consequently prescribed darafenib plus trametinib targeted BRAF V600E, but 3 months later, increased metastases appeared in both lungs, and massive hemoptysis occurred in March 2020, indicating tumor progressing. Darafenib plus trametinib targeted therapy was continued and supplemented by 3 cycles of chemotherapy. Although brain metastases decreased, pleural effusion increased and liver metastasis appeared in May 2020. We thus challenged the patient’s anti-tumor regimen and then prescribed ALK inhibitor alectinib 600 mg twice one day in June 2020 after written informed consent obtained. The disease had a rapid response to alectinib. Close follow-up revealed that a complete response of widespread metastatic including lung lesion, adrenal gland, liver and brain, and a significant partial response (PR) in the size of lymph node metastases, along with the carcinoembryonic antigen returning to the normal range, as shown in Figure 1. The last follow-up was conducted in August 2022, and the patient has a progression-free survival (PFS) more than 26 months with continuous administration of alectinib with none severe adverse reactions observed.

**Figure 2.**
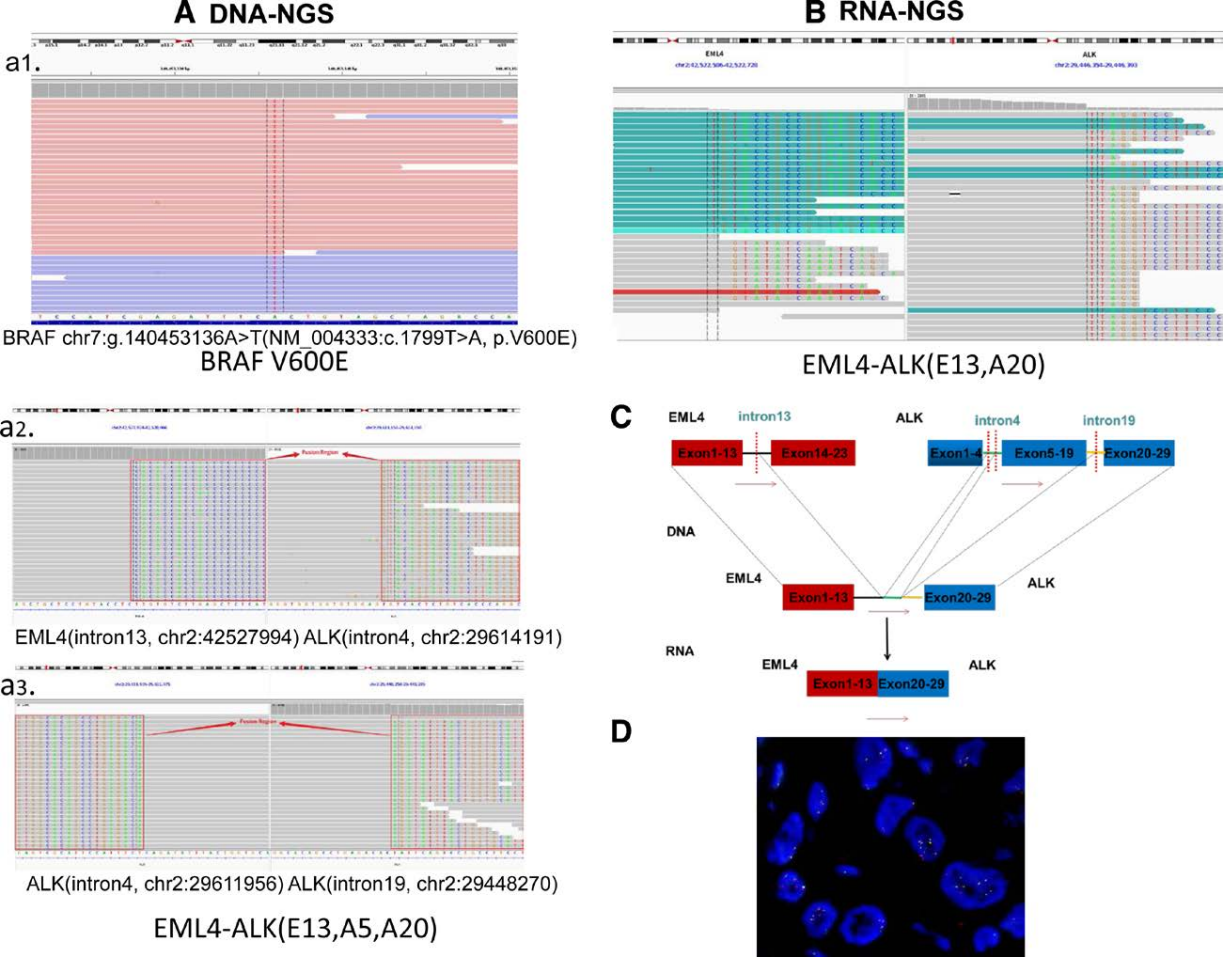
(A) IGV diagram of DNA-NGS BRAF V600E mutation and EML4 (intron13)-ALK (intron4)-ALK (intron19) complex fusion. (B) RNA-NGS EML4-ALK (E13, A20). (C) Complex fusion mockup. (D) ALK FISH Assay. ALK = anaplastic lymphoma kinase, FISH = fluorescence in situ hybridization, NGS = next-generation sequencing.

We were surprised by the patient’s good response. To validate the clinical significance of the novel fusion isoform EML4-ALK (E13, A5), we performed fluorescence in situ hybridization on the patient’s lymph node biopsy and verified the presence of the ALK fusion in Jul 2022. We also analyzed the patient’s DNA-NGS original data again and found that was a rare non-classical EML4 (intron13)-ALK (intron4)-ALK (intron19) complex fusion in fact, shortened to EML4-ALK (E13, A5, A20). We performed RNA-NGS (Target RNA sequencing) on the lymph node biopsy and found that the complex EML4-ALK fusion at the DNA level actually verified to be a canonical EML4-ALK (E13, A20) fusion transcript at the RNA level, and the structure was shown schematically in Figure 2.

## 3. Discussion

Advances in ALK-positive NSCLC therapy depend on the improvement of detection technology of ALK rearrangements. The prevenient detection techniques such as fluorescence in situ hybridization and immunohistochemistry are unable to identify novel ALK fusion partners or resolve complex structural rearrangements, but NGS can.^[4,5]^ With the widespread application of NGS, >90 ALK fusion partners have been identified in NSCLC.^[6]^ EML4-ALK is the most frequent fusion variant of ALK rearrangements. To date, the chromosomal breakpoint within the EML4 gene occurs at different sites, such as exons 2, 3, 6, 7, 8, 10, 13, 14, 15, 16, 17, 18, 19, 20, 21, and 23, while the breakpoint within ALK was found most frequently at exon 20, occasionally at exon18/19.^[7,8]^

Complex EML4-ALK fusion at DNA level is not common but reported in few studies. In the cohort study directed by Peiyi Xia et al,^[9]^ complex EML4-ALK rearrangements were identified in 13 cases among 343 cases positive for ALK fusion, as detected by DNA-NGS, and one of them featured the rare and complicated intragenic rearrangement of ALK gene EML4-ALK (E6, A5, A20), similar to this case. Unfortunately, there were not enough specimens for RNA level verification in that patient. This case we reported makes up for their regret. However, it was generated to be a canonical EML4-ALK (E13, A20) fusion transcript at RNA level. As has been reported in the study of Weihua Li et al,^[10]^ RNA-NGS showed that 6 of 47 (12.8%) uncommon fusions at DNA level actually were nonproductive arrangements that generated no aberrant transcripts or proteins. The diversity of ALK fusion breakpoints at the DNA level and their inconsistent expression at the RNA level have attracted our attention. Exclusively reliance on rare fusion breakpoints detected by DNA-NGS for optimal treatment decision and predicting the efficacy of targeted therapy may lead to inaccuracy. Mechanically,^[11,12]^ fusion breakpoints at the DNA level usually occur in long intron regions so that probes detecting fusion genes have to cover very long intron regions containing a large number of repetitive sequences in order to sensitively position fusion breakpoints at the DNA level, leading to insufficient detection rate and misinterpretation. The fusion gene at the RNA level is represented by the articulation between the exons of the two genes before and after the fusion. The fusion sites at the RNA level are relatively more accurate. For common EML4-ALK positive NSCLC, crizotinib, ceritinib or alectinib are recommended as first-line treatment in the NCCN Guidelines.^[13]^ Whether patients harboring complex genomic rearrangements could benefit differently from variant ALK tyrosine kinase inhibitors is unclear. Peiyi Xia et al found no significant difference in median PFS between the patients carrying complex and canonical ALK fusions regardless of their first-line treatment, crizotinib or alectinib,^[9]^ while Weihua Li et al reported that complex ALK fusion showed a significantly shorter PFS when treated with crizotinib than alectinib.^[10]^ This case, with a PFS more than 26 months, indicated that complex ALK fusion at DNA level did not affect the effect of alectinib. Limited by sample size, the conclusion requires more cohort studies and future endeavors for verification.

To our surprise, this case harbored a rare co-mutation of EML4-ALK fusion and BRAF V600E prior to treatment. BRAF mutations have previously been detected in ALK-positive NSCLC patients after crizotinib and ceritinib treatment, and researchers argued that BRAF V600E may be a downstream pathway of ALK and cause resistance to ALK inhibitors.^[14,15]^ To our knowledge, this is the first successfully cured patient who harbored coexistence of ALK and BRAF V600E prior to treatment; A similar case of squamous cell carcinoma who died before receiving crizotinib was reported by D. ALrifai.^[16]^ Whether the co-mutation affects the choice of tyrosine kinase inhibitors drugs is still not clearly established. Crizotinib or alectinib are recommended as first-line treatment for ALK positive NSCLC,^[13,17]^ while darafenib plus trametinib was recommended for BRAF V600E positive patients in the NCCN Guidelines.^[13,18]^ Complexity of the patient’s NGS at the DNA level resulted a misjudgment and targeted BRAF V600E were prescribed firstly before target EML4-ALK; however, it provides a chance to distinguish which oncogenic signaling pathway is more important for lung cancer. PFS of this case taking darafenib plus trametinib was 5 months, which is much lower than the median PFS in previous clinical trial (10.9 mo) enrolling patients with untreated BRAF V600E-mutant metastases.^[19]^ The fact that this case responded poorly to darafenib plus trametinib but well to alectinib, indicating that ALK activation, rather than BRAF V600E, is the main driver factor of lung cancer when they coexist. Moreover, long PFS of more than 26 months of the patient suggests that coexisting with BRAF V600E will not affect the efficacy of ALK inhibitors. To sum up, targeted EML4-ALK firstly may be more preferable despite the coexisting with BRAF V600E.

## 4. Conclusion

In conclusion, for rare or complex EML4-ALK fusions at DNA level, additional RNA-NGS is necessary to verify its functionality, and EML4-ALK-targeted therapy may be more preferable despite coexisting of BRAF V600E.

## Author contributions

**Conceptualization:** Weihong Guo, Yanhua Lv.

**Data curation:** Weihong Guo.

**Formal analysis:** Yanhua Lv.

**Investigation:** Jianping Liang.

**Methodology:** Dandan Zhang.

**Software:** Xikun Huang.

**Writing – original draft:** Weihong Guo, Yanhua Lv.

**Writing – review & editing:** Weihong Guo, Yanhua Lv.
